# DEVELOPING A MUSIC PROGRAM ON ZOOM FOR OLDER ADULTS WITH DECLINING COGNITION: A PROGRAM DEVELOPMENT REPORT

**DOI:** 10.1093/geroni/igac059.177

**Published:** 2022-12-20

**Authors:** Jennie Dorris, Heather DiCicco, James Becker, Juleen Rodakowski

**Affiliations:** University of Pittsburgh, Pittsburgh, Pennsylvania, United States; BriTE Wellness Inc, Pittsburgh, Pennsylvania, United States; University of Pittsburgh, Pittsburgh, Pennsylvania, United States; University of Pittsburgh, Pittsburgh, Pennsylvania, United States

## Abstract

The coronavirus pandemic upended in-person programming for older adults with cognitive decline, presenting an urgent need to explore options to offer virtual programming. This program development report describes the adaptation of a music program for older adults with memory loss from in-person to a digital format. The objective was to develop a music program that was both engaging for the older adults, acceptable for the music instructor, and clearly defined for future research and implementation. This report describes the content of the music program and the systematic process of its development, including stakeholder interviews, acceptability surveys, fidelity reviews, and the developed instructor manual. With these structures in place, future research could begin to discern if digital music programming can support older adults in the same way that has been demonstrated in-person, potentially offering a new way to support the cognitive and social needs of a vulnerable population.

